# Trends in Norwegian adolescents’ substance use between 2014 and 2022: socioeconomic and gender differences

**DOI:** 10.1186/s12889-024-19983-9

**Published:** 2024-09-12

**Authors:** Arnhild Myhr, Renate K. Vesterbekkmo, Indira Samarawickrema, Erik R. Sund

**Affiliations:** 1 Department of Technology Management, SINTEF Digital, Steinkjer, 7715 Norway; 2 Regional Drug and Alcohol Competence Centre, St. Olavs University Hospital, Trondheim, Norway; 3 Faculty of Health, University of Canberra, Canberra, ACT Australia; 4 Department of Public Health and Nursing, Faculty of Medicine and Health Sciences, HUNT Research Centre, Norwegian University of Science and Technology, Levanger, Norway; 5 Levanger Hospital, Nord-Trøndelag Hospital Trust, Levanger, Norway

**Keywords:** Alcohol intoxication, Cannabis, Illicit drugs, Time trends, Socioeconomic inequalities, Adolescents, Gender, Norway

## Abstract

**Background:**

Substance use is a global health concern and early onset among adolescents increases health risks. We explore national overall trends in prevalence and trends in socioeconomic inequalities in past year alcohol intoxication, cannabis use, and use of other illicit drugs among Norwegian adolescents (ages ∼ 15–19 years of age) between 2014 and 2022.

**Method:**

The present study builds on data from a nationwide repeated cross-sectional survey collected in 2014–2016 (T1), 2017–2019 (T2), 2021 (T3) and 2022 (T4). In total 415,560 adolescents (50.3% girls) completed the questionnaire during the study period. Trends in socioeconomic inequalities were assessed using the Slope Index of Inequality (SII) and the Relative Index of Inequality (RII).

**Results:**

While the prevalence of alcohol intoxication remained fairly stable, the prevalence of cannabis and other illicit drug use increased between 2014 and 2022 among upper secondary school boys (13.3–17.6%, and 2.0–5.2%, respectively) and girls (8.8–12.8%, and 1.1–2.7%, respectively). Similar trends were observed among 10th-grade adolescents. Boys were more likely than girls to use cannabis or other illicit drugs, but the gender gap in cannabis use narrowed during the study period. Among upper secondary girls, use of cannabis and other illicit drugs was higher among those from less affluent backgrounds, with absolute and relative inequalities in cannabis use increasing between 2014 and 2022. Small inequalities in cannabis use and decreasing relative inequalities in the use of other illicit drug were observed among upper secondary boys.

**Conclusions:**

The increasing use of cannabis and other illicit drugs among Norwegian adolescents is concerning. Future studies should explore the underlying causes of this rise and explore the complex factors influencing adolescent substance use behaviours. A comprehensive understanding of these factors is essential for developing targeted and effective interventions.

**Supplementary Information:**

The online version contains supplementary material available at 10.1186/s12889-024-19983-9.

## Introduction

Substance use is a leading global cause of premature death and disability-adjusted life years among young people [1, 2]. In Norway, the social costs from alcohol use in 2022 were estimated to reach a total of USD 9.4 billion, encompassing healthcare expenses, lost productivity, and social welfare expenditures [3]. According to the latest data from the European Survey Project on alcohol and other drugs (ESPAD) [4], there has been a decline in cigarette smoking and alcohol consumption among adolescents overt the past decades. However, the use of cannabis and other illicit drugs has remained stable or increased. These findings are supported by the recent European drug report [5], although significant variations on substance use patterns across different countries and demographic groups exists. Substance use among adolescents is a public health concern as early onset increases the risk of substance use disorders, dependence, and poorer psychological, social, and physical health [6]. With the rising prevalence of adolescent substance use observed across several European countries [7], a deeper understanding of this burden is necessary for effective preventive strategies. In this context, representative population-based studies are essential to assess adolescents’ substance use patterns over time, determining their magnitude and fluctuations.

Adolescence is characterized by risky decision-making and heightened sensation seeking, making it a peak time for substance initiation, often whit alcohol preceding illicit drugs use [2, 8]. Substance use patterns among adolescents vary between countries due to social contexts, drug availability, and personal traits [2, 9]. In Europe, alcohol consumption often initiate between ages of 12 and 16, with higher rates observed in high-income countries [10]. Among Norwegian 15 to 16 year-olds, alcohol use prevalence is relatively low compared to many other European countries, however, drinking frequency escalates notably with age [4]. Binge or heavy episodic drinking is particularly prevalent among 15 to 19-year-olds in Europe [10]. Consequently, these young people are at increased risk of negative experiences such as violence, unwanted sexual encounters, accidents, and physical injuries [11].

Globally, alcohol and illicit drug use is more prevalent among males, although the gender gap narrows among younger cohorts [4]. This gap tends to be wider in poorer communities compared to more affluent ones within societies [10]. The illicit drug availability and use persist at significant levels across the EU, although considerable variations exist both between and within countries [7, 12]. Cannabis, which remains illegal in Norway, is the most commonly used illicit drug, followed by stimulants such as cocaine, amphetamine and MDMA [13]. Although Norwegian adolescents use cannabis to a lesser extent than their European peers, the prevalence has increased since the mid-2010s [4, 14].

While it is well-established that low socioeconomic position (SEP) is associated with higher risk for hazardous health behavior [15], the evidence regarding SEP and adolescents’ substance use is mixed or inclusive [16, 17]. Nevertheless, at the population level, there is a clear trend of higher alcohol consumption in economically affluent countries and areas, and among more affluent population groups [10, 18–20]. Although alcohol consumption is more likely to be higher in more affluent groups, alcohol-related morbidity and mortality are more common in more deprived areas and in groups with lower SEP, especially among younger birth cohorts [21, 22]. Lower SEP adolescents are also found to be at higher risk of early, frequent and heavy drinking compared to their higher SEP peers [23]. According to Spooner & Hetherington [9], the association of SEP with adolescents illicit drug varies according to context, setting and substance. In recent years, European and American studies have shown that cannabis use is more common among higher SEP adolescents [24–26]. A French study found that while affluent adolescents were more likely to experiment with cannabis, less affluent adolescents were more likely to engage in high-level use [25]. These findings are supported by other studies, findings that high SEP adolescents are more likely to ever try cannabis, but that low SEP adolescents are at higher risk of frequent or problematic drug use [27, 28]. Evidence suggest that low SEP adolescents also are more at risk of multiple substance use [29, 30]. Moreover, a recent Norwegian study found that multiple substance use was associated with increased risk in various life domains, including low parental control, mental health issues, and conduct problems [30].

Initial findings following the COVID-19 pandemic suggest a deterioration in adolescents’ general well-being and life satisfaction [31–34], accompanied by changes in patterns of substance use [35, 36]. Although many adolescents experienced transitory distress during and following the pandemic, evidence suggests that those of low SEP, girls and younger adolescents, exhibited more adverse psychosocial changes than other groups in the population [33, 34]. The pandemic may also have accelerated digitalization within the drugs market, promoting the use of social media platforms as arenas for drug dealing. The use of such platforms could make illegal substances more accessible to adolescents, potentially sparking their curiosity and leading to initial experimentation [37]. Moreover, increased access may also lead to the extended use of a variety of different substances [38].

While adolescent substance use generally fluctuates over time, it can be hypothesized that the pandemic influenced behavior patterns. Numerous studies have examined adolescent substance use during the pandemic, and a recent systematic review concluded that, for the most part, substance use among adolescents decreased during the outbreak, with the exception of small increases in unspecified drugs [39]. To the best of our knowledge, only one other population-based study has focused on adolescent substance use two years after the first lockdown [40]. This study found that alcohol intoxication initially decreased during the pandemic but increased again as social restrictions eased. However, the study did not examine the use of illicit drugs or social inequalities in such use.

This study uses large-scale population data from eight waves of the Ungdata survey (*n* = 415,560) to examine overall trends and changes in absolute and relative inequalities in Norwegian adolescent substance use, including alcohol intoxication and the use of cannabis and other illicit drugs between 2014 and 2022.

## Methods

### Study design and participants

The present study is based on data from the Norwegian nationwide Ungdata surveys. Ungdata is considered the most wide-ranging source of data on health and well-being among adolescents attending lower (aprox. aged 13 to 15) and upper (aprox. aged 16 to 18) secondary education in Norway. Adolescents in nearly all municipalities are regularly assessed, typically every third year. The survey is conducted electronically during school hours, with participation being voluntary and based on informed consent by students. Parents or guardians can opt their children out of participation. The survey is administrated by the NOVA Welfare Research Institute based at Oslo Metropolitan University (OsloMet), in collaboration with Norwegian regional drug and alcohol competence centers (KORUS).

In this study, we included all data available from 2014 to 2022 among adolescents in their final year of compulsory lower secondary education (10th-grade) and those in upper secondary school (first to third year students). Given that substance use is relatively uncommon among the youngest Norwegian adolescents [41] we focused on adolescents in their final year of lower secondary school and those in upper secondary school. In 2020, only a few municipalities were able to conduct the Ungdata survey before school closures due to the COVID-19 pandemic, so this data was excluded from our analysis. Response rates were consistently high, ranging from the lowest at 75% in the period 2014 to 2016, to a peak of 78% in 2017 [42–47]. Rates were higher among the lower secondary compared to the upper secondary students.

The full study sample contained 451,960 adolescents which was reduced due to missing information on gender (*n* = 13,886), school year (*n* = 16,248) and family SEP (*n* = 6,266) rendering a sample of 415,560 respondents. Due to missing information related to respondents’ past-year intoxication (*n* = 15,338), cannabis use (*n* = 15,448) and use of other illicit drugs (*n* = 23,064) sample sizes thus varies between outcomes (valid sample sized are reported in result tables). Also, 10th-grade students were only asked whether they had consumed ‘*other illicit drugs’* in the 2021 and 2022 surveys.

Since most municipalities conduct the Ungdata survey every three years, we aggregated data from surveys conducted over three-year periods to create a representative national database. We divided our dataset into four time periods to ensure representativeness: (i) 2014–2016 (T1), (ii) 2017–2019 (T2), (iii) 2021 (T3) and (iv) 2022 (T4). The data for periods T3 and T4 are limited to data collected in 2021 and 2022, respectively, as these years represent distinct phases of the pandemic. Data from 2021 reflects the period after more than a year of ongoing pandemic, during which many areas experienced extensive restrictions. Data from 2022 corresponds to the period following the easing of all restrictions as society began to return to normal.

### Measures

#### Outcome measures

The outcome variables we analyzed in this study were *past-year alcohol intoxication*,* cannabis use*,* and use of other illicit drugs*, and were addressed using the following questions; How many times during the past 12 months have you (i) consumed enough alcohol to feel intoxicated; (ii) used hash/marijuana/cannabis; (iii) used other illicit drugs?

The response categories were coded as follows; (1) Never, (2) Once, (3) 2 to 5 times, (4) 6 to 10 times, (5) eleven or more times. We constructed dichotomous variables in order to distinguish between respondents reporting no past-year experiences (coded ‘0’), from those reporting one or more past-year experiences of ‘alcohol intoxication’, ‘cannabis use’ or ‘use of other illicit drugs’ (coded ‘1’).

#### Socioeconomic position (SEP)

Adolescent SEP was measured using a collective measure of SEP developed by Bakken and colleagues [48] which includes, in addition to the four-point instrument Family Affluence Scale (FAS) II [49, 50], information in parental education levels, and the numbers of books in the home. The FAS II instrument elicited the number of cars, computers and/or tablets in the family, the number of annual holidays, and whether respondents had their own bedroom [49]. In order to protect respondent anonymity, *Ungdata* surveys do not include questions about parents’ occupations or incomes. Results from the FAS II scale have been validated alongside other measures of adolescent SEP. It has been found that this scale exhibits better criterion validity and less susceptibility to non-response bias compared with measures based on parental income, occupation or education levels [51]. The calculated mean sum score, ranging from 0 to 3, was split into five equally sized groups, ranging from the highest to lowest SEP.

#### Covariates

Substance use and experience tend to increase with age [52], and therefore, age was included as a covariate in our main analysis. School grade, serving as a proxy for age, was categorized as follows: elementary 10th-grade (approximately 15 to 16 years old), and first, second, and third year of upper secondary school (approximately 16 to 19 years old).

### Statistical analysis

The prevalence of past-year intoxication, cannabis use, and other illicit drug use was calculated for each gender (and separately for elementary 10th-grade and upper secondary students) and adjusted for the respondent’s school grade. Slope index of inequality (SII) and relative index of inequality (RII) were calculated to investigate socioeconomic inequalities in substance use [53–55]. Specifically, each SEP category was assigned a ridit score based on the mid-point of the range in a cumulative distribution of the population of respondents. Using generalized linear models (GLM) with logarithmic and identity link functions, the ridit score was regressed on the outcomes yielding RII and SII respectively. We examined trends in RII and SII over time by pooling the four time periods and including a two-way interaction term between the ridit-score and time. In terms of interpretation, values of SII > 0 and RII > 1 indicate that the outcome is more prevalent among adolescents with a lower SEP than those at a higher position.

We conducted supplementary analyses to explore potential gender differences and trends in such differences in adolescents’ substance use applying logistic regression and the inclusion of a two-way interaction term between gender and time. Since evidence suggest that Norwegian adolescents living in major cities have higher cannabis use compared to their peers in more sparsely populated areas [52], we also performed stratified analyses based on whether the respondent resides in a major municipality or not.

All analyses were performed on complete cases and estimates are reported with 95 per cent confidence intervals (95% CI). Statistical analyses were conducted using Stata version 17 (StataCorp LLC, College Station, Texas, USA).

## Results

### Sample characteristics

Table 1 presents the characteristics of the study sample, stratified by gender and time. Among boys, the prevalence of past-year alcohol intoxication increased from T1 to T2 (42.3–47.4%) but decreased during and after the pandemic at T3 (43.7%) and T4 (42.2%). Among girls, the prevalence of alcohol intoxication slightly increased from 46.4 to 49.8% between 2014 and 2022. During this period, a small increase in past-year cannabis and other illicit drug use was observed among boys (from 10.2 to 14.3% and 1.9–4.3%, respectively) and girls (from 6.7 to 10.6% and 1.1–2.4%, respectively). Cannabis and other illicit drug use were higher among boys, while alcohol intoxication was more prevalent among girls.

**Table 1:** Unadjusted characteristics of study sample (*n* = 415,560) by gender and time period in the Ungdata survey (2014–2022)

	Boys				Girls			
	T1 (2014–2016) (*n* = 51,287)	T2 (2017–2019) (*n* = 86,040)	T3 (2021) (*n* = 38,613)	T4 (2022) (*n* = 30,669)	T1 (2014–2016) (*n* = 51,258)	T2 (2017–2019) (*n* = 86,736)	T3 (2021) (*n* = 40,492)	T4 (2022) (*n* = 30,465)
**Sociodemographic characteristics**								
School year								
10th grade lower secondary	36.5	29.7	33	33.5	35.6	29.2	31.4	32
1st upper secondary	33	31.5	29.6	30	31.9	29.7	27.1	28.8
2nd upper secondary	21.3	25	24.3	25.5	20.4	23.8	23.3	24.9
3rd upper secondary	9.3	13.8	13.1	11	12.1	17.2	18.2	14.3
Resident in major municipality	17.7	22.7	24.7	13.9	19.4	23.7	26	14.8
SEP (mean SD)	1.99	2.01	1.95	1.99	1.99	2.03	2	2.03
**Substance use in the past year**								
Alcohol intoxication	42.3	47.4	43.7	42.2	46.4	49.3	50.2	49.8
Cannabis use	10.2	15.2	12.9	14.3	6.7	8.8	8.9	10.6
Use of other illicit drugs	1.9	3.7	3.7	4.3	1.1	1.7	1.8	2.4

Tables 2, 3, 4 and 5 in the following sections present the prevalence, Relative Index of Inequality (RII), and Slope Index of Inequality (SII) of past-year alcohol intoxication, cannabis use, and the use of other illicit drugs among boys and girls in 10th-grade and upper secondary education between 2014 and 2022.

### Alcohol intoxication

The prevalence of alcohol intoxication among 10th-grade boys decreased slightly between 2014 and 2022 (from 22.8 to 20.7%, *p* < 0.001), while it increased slightly among girls (25.7–28.1%, *p* < 0.001). Alcohol intoxication was more prevalent among lower SEP boys and girls compared to their higher SEP peers, and these inequalities remained stable in both absolute and relative terms over the study period. A test for gender differences (Table S1 in supplementary materials) revealed that past-year intoxication was higher among girls than boys, and this gender gap widened over the study period (Odds Ratios: 1.18 to 1.50, *p* < 0.001).

Among upper secondary students, the prevalence of alcohol intoxication decreased slightly among boys (from 55.3 to 53.3%, *p* < 0.001), while it remained stable at about 60% among girls during the study period. In contrast to the 10th-grade students, alcohol intoxication was more prevalent among upper secondary boys and girls with a higher SEP compared to their lower SEP peers. Among boys, the results suggest decreases in SEP inequalities in both relative (from 0.88 to 0.92, *p* = 0.006) and absolute (from − 0.06 to -0.04, *p* = 0.007) terms. Past-year intoxication (Table S2 in supplementary materials) was higher among girls compared to boys, and this trend intensified slightly over the study period (Odds ratios: 1.15 to 1.29, *p* < 0.001).

### Cannabis use

Past-year use of cannabis slightly increased among 10th-grade boys (from 5.5 to 8.1%, *p* < 0.001) and girls (3.2–6.0%, *p* < 0.001). Prevalence`s decreased slightly at T3 but rose again at T4. Cannabis use was more prevalent among lower SEP boys and girls opposed to their lower SEP peers, and both absolute and relative inequalities remained relatively stable during the study period. Girls were less likely than boys to have used cannabis in the past year (Table S1 in supplementary materials), but the gender gap narrowed over the study period (Odds Ratios: 0.57 to 0.74, *p* = 0.012).

Cannabis use also increased in upper secondary boys (from 13.3 to 17.6%, *p* < 0.001) and girls (8.8–12.8%, *p* < 0.001) between 2014 and 2022. Among boys, cannabis use was slightly higher in those with higher SEP compared to those with lower SEP, although relative inequalities decreased slightly during this period (from 0.84 to 1.05, *p* < 0.001). Among girls, cannabis use was more prevalent among those with lower SEP opposed to their higher SEP peers and inequalities increased in both absolute (from 0.00 to 0.05, *p* < 0.001) and relative (from 0.92 to 1.51, *p* < 0.001) terms. Girls were less likely than boys to have used cannabis (Table S2 in supplementary materials), although the gender gap slightly narrowed from 2014 to 2022 (Odds Ratios: 0.61 to 0.67, *p* = 0.007).

### Use of other illicit drugs

Past year use of other illicit drugs was fairly stable between T3 to T4 among 10th-grade boys (from 2.3 to 2.5%) and girls (from 1.4 to 1.7%). The use of other illicit drugs were more prevalent among lower SEP individuals and absolute inequalities slightly increased among boys (from 0.01 to 0.02, *p* < 0.001). Boys were more likely than girls to use other illicit drugs during the past year (Table S1 in supplementary material).

The use of other illicit drugs also increased from T1 to T4 among boys (from 2.0 to 5.2%, *p* < 0.001) and girls (1.1–2.7%, *p* < 0.001) attending upper secondary school. Prevalence was highest among lower SEP boys and girls compared to their higher SEP peers. Among girls, the results suggest a small increase in absolute inequalities (SII: 0.01 to 0.02, *p* < 0.001) from T1 to T4. In boys, a decrease in relative inequalities was observed (RII: 2.04 to 1.21, *p* = 0.011). Boys were consistently more likely than girls to use other illicit drugs during the past year, and this gender difference remained stable during the study period (Table S2 in supplementary materials).

**Table 2:** Prevalence (%), absolute and relative SEP inequalities in past-year alcohol intoxication, cannabis use and use of other illicit drugs* among boys in 10th-grade between 2014 and 2022 as reported in the Ungdata survey

	Alcohol intoxication (*n* = 64,560)			Cannabis use (*n* = 64,479)			Other illicit drugs (*n* = 23,418)		
	Coeff.	95% CI		Coeff.	95% CI		Coeff.	95% CI	
		Lower	Upper		Lower	Upper		Lower	Upper
**Prevalence**									
T1 (2014–2016)	22.8	22.2	23.4	5.5	5.2	5.9			
T2 (2017–2019)	23.6	23.1	24.2	8.0	7.7	8.4			
T3 (2021)	21.5	20.8	22.3	6.8	6.3	7.2	2.3	2.0	2.5
T4 (2022)	20.7	19.9	21.5	8.1	7.6	8.6	2.5	2.2	2.8
p-value for trend	*p* < 0.001			*p* < 0.001			*p* = 0.130		
Relative index of inequality^a^									
T1 (2014–2016)	1.17	1.06	1.29	1.58	1.27	1.97			
T2 (2017–2019)	1.10	1.01	1.19	1.35	1.16	1.57			
T3 (2021)	1.17	1.04	1.32	1.59	1.25	2.01	2.80	1.82	4.29
T4 (2022)	1.24	1.07	1.43	1.81	1.41	2.31	2.23	1.41	3.54
p-value for trend	*p* = 0.484			*p* = 0.237			*p* = 0.518		
Slope index of inequality^b^									
T1 (2014–2016)	0.04	0.01	0.06	0.02	0.01	0.03			
T2 (2017–2019)	0.02	0.00	0.04	0.02	0.01	0.03			
T3 (2021)	0.03	0.01	0.06	0.03	0.01	0.04	0.01	0.00	0.01
T4 (2022)	0.04	0.01	0.07	0.05	0.03	0.07	0.02	0.01	0.03
p-value for trend	*p* = 0.580			*p* = 0.048			*p* = 0.001		

Data are regression-based predicted means (95% CI)* adjusted for differences in the respondents’ school year (^a^) represents the prevalence-ratio for substance use between the lowest and highest ranked families. (^b^) represents the risk difference for substance use between the least and most affluent families

**Table 3:** Prevalence (%), absolute and relative inequalities in past-year alcohol intoxication, cannabis use and use of other illicit drugs* among girls in 10th-grade between 2014 and 2022 as reported in the Ungdata survey

	Alcohol intoxication (*n* = 64,824)			Cannabis use (*n* = 64,752)			Other illicit drugs (*n* = 23,581)		
	Coeff.	95% CI		Coeff.	95% CI		Coeff.	95% CI	
		Lower	Upper		Lower	Upper		Lower	Upper
**Prevalence**									
T1 (2014–2016)	25.7	25.1	26.4	3.2	3.0	3.5			
T2 (2017–2019)	24.9	24.4	25.4	4.4	4.2	4.7			
T3 (2021)	26.1	25.4	26.9	4.1	3.8	4.5	1.4	1.2	1.6
T4 (2022)	28.1	27.2	29.0	6.0	5.5	6.5	1.7	1.5	2.0
p-value for trend	*p* < 0.001			*p* < 0.001			*p* = 0.026		
Relative index of inequality^a^									
T1 (2014–2016)	1.33	1.22	1.45	3.40	2.53	4.56			
T2 (2017–2019)	1.22	1.13	1.32	2.95	2.40	3.62			
T3 (2021)	1.07	0.96	1.18	2.01	1.50	2.71	2.16	1.29	3.62
T4 (2022)	1.20	1.07	1.35	2.66	2.00	3.54	3.58	2.06	6.25
p-value for trend	*p* = 0.027			*p* = 0.087			*p* = 0.190		
Slope index of inequality^b^									
T1 (2014–2016)	0.07	0.05	0.09	0.04	0.03	0.04			
T2 (2017–2019)	0.05	0.03	0.07	0.04	0.03	0.05			
T3 (2021)	0.02	-0.01	0.04	0.03	0.02	0.04	0.01	0.00	0.02
T4 (2022)	0.05	0.02	0.08	0.06	0.04	0.07	0.02	0.01	0.03
p-value for trend	*p* = 0.048			*p* = 0.087			*p* = 0.048		

Data are regression-based predicted means (95% CI)* adjusted for differences in the respondents’ school year (^a^) represents the prevalence-ratio for substance use between the lowest and highest ranked families. (^b^) represents the risk difference for substance use between the least and most affluent families

**Table 4:** Prevalence (%), absolute and relative inequalities in past-year alcohol intoxication, cannabis use and use of other illicit drugs* among boys in upper secondary education between 2014 and 2022 as reported in the *Ungdata* survey

	Alcohol intoxication (*n* = 130,371)			Cannabis use (*n* = 130,303)			Other illicit drugs (*n* = 125,038)		
	Coeff.	95% CI		Coeff.	95% CI		Coeff.	95% CI	
		Lower	Upper		Lower	Upper		Lower	Upper
**Prevalence**									
T1 (2014–2016)	55.3	54.8	55.8	13.3	12.9	13.7	2.0	1.8	2.1
T2 (2017–2019)	57.2	56.8	57.6	18.2	17.8	18.5	3.7	3.5	3.8
T3 (2021)	54.2	53.5	54.8	15.8	15.3	16.3	4.3	4.0	4.5
T4 (2022)	53.3	52.7	54.0	17.6	17.1	18.2	5.2	4.9	5.5
p-value for trend	*p* < 0.001			*p* < 0.001			*p* < 0.001		
Relative index of inequality^a^									
T1 (2014–2016)	0.88	0.85	0.91	0.84	0.76	0.93	2.04	1.53	2.74
T2 (2017–2019)	0.89	0.87	0.91	0.83	0.78	0.88	1.44	1.23	1.69
T3 (2021)	0.94	0.91	0.98	0.89	0.80	0.98	1.41	1.14	1.74
T4 (2022)	0.92	0.89	0.97	1.05	0.94	1.17	1.21	0.97	1.50
p-value for trend	*p* = 0.006			*p* < 0.001			*p* = 0.011		
Slope index of inequality^b^									
T1 (2014–2016)	-0.06	-0.08	-0.04	-0.01	-0.03	0.00	0.01	0.01	0.02
T2 (2017–2019)	-0.06	-0.08	-0.05	-0.03	-0.04	-0.02	0.01	0.01	0.02
T3 (2021)	-0.02	-0.04	0.00	-0.02	-0.03	0.00	0.02	0.01	0.02
T4 (2022)	-0.04	-0.06	-0.01	0.01	-0.01	0.03	0.01	0.00	0.02
p-value for trend	*p* = 0.007			*p* = 0.069			*p* = 0.151		

Data are regression-based predicted means (95% CI)* adjusted for differences in the respondents’ school year (^a^) represents the prevalence-ratio for substance use between the lowest and highest ranked families. (^b^) represents the risk difference for substance use between the least and most affluent families

**Table 5:** Prevalence (%), absolute and relative inequalities in past-year alcohol intoxication, cannabis use and use of other illicit drugs* among girls in upper secondary education between 2014 and 2022 in the *Ungdata* survey

	Alcohol intoxication (*n* = 138,200)			Cannabis use (*n* = 138,216)			Other illicit drugs (*n* = 132,429)		
	Coeff.	95% CI		Coeff.	95% CI		Coeff.	95% CI	
		Lower	Upper		Lower	Upper		Lower	Upper
**Prevalence**									
T1 (2014–2016)	59.5	59.0	60.0	8.8	8.5	9.2	1.1	1.0	1.2
T2 (2017–2019)	59.2	58.8	59.6	10.6	10.3	10.8	1.7	1.5	1.8
T3 (2021)	60.2	59.6	60.7	10.8	10.5	11.2	1.9	1.8	2.1
T4 (2022)	60.4	59.7	61.1	12.8	12.4	13.3	2.7	2.5	3.0
p-value for trend	*p* = 0.004			*p* < 0.001			*p* < 0.001		
Relative index of inequality^a^									
T1 (2014–2016)	0.89	0.86	0.91	0.92	0.81	1.04	2.81	1.92	4.12
T2 (2017–2019)	0.91	0.90	0.93	1.05	0.96	1.14	2.24	1.77	2.84
T3 (2021)	0.93	0.90	0.95	1.20	1.07	1.36	2.31	1.71	3.12
T4 (2022)	0.92	0.88	0.95	1.51	1.33	1.71	2.23	1.66	2.99
p-value for trend	*p* = 0.091			*p* < 0.001			*p* = 0.472		
Slope index of inequality^b^									
T1 (2014–2016)	-0.06	-0.08	-0.04	0.00	-0.01	0.01	0.01	0.01	0.01
T2 (2017–2019)	-0.05	-0.06	-0.04	0.01	0.00	0.02	0.01	0.01	0.02
T3 (2021)	-0.04	-0.06	-0.02	0.02	0.01	0.03	0.02	0.01	0.02
T4 (2022)	-0.05	-0.07	-0.03	0.05	0.04	0.07	0.02	0.01	0.03
p-value for trend	*p* = 0.256			*p* < 0.001			*p* < 0.001		

Data are regression-based predicted means (95% CI)* adjusted for differences in the respondents’ school year (^a^) represents the prevalence-ratio for substance use between the lowest and highest ranked families. (^b^) represents the risk difference for substance use between the least and most affluent families

### Secondary analyses

In the stratified analyses (Tables S3–S10 in supplementary materials), the prevalences of alcohol intoxication, cannabis use and the use of other illicit drugs tended to be higher among individuals residing in major municipalities compared to those living in more sparsely populated areas. This pattern was consistent across gender and school grade levels. Among upper secondary adolescents, the relative risk of cannabis use was higher among higher SEP adolescents in major municipalities, whereas the relative risk was higher among lower SEP individuals in more sparsely populated areas.

## Discussion

In this large population-based study, we observed an increase in the prevalences of past year use of cannabis and other illicit drugs among boys and girls in upper secondary school between 2014 and 2022. The use of cannabis also increased among 10th-grade adolescents. Alcohol intoxication prevalence remained relatively stable over the same period. While the alcohol intoxication was higher among girls, cannabis and other illicit drug use was higher among boys. Illicit drug use also increased among girls, narrowing the gender gap in cannabis use during the study period. 10th-grade adolescents from less affluent backgrounds showed a higher substance use prevalence, while results were more mixed for upper secondary school students. Among girls, both relative and absolute inequalities in cannabis use increased during the study period, whereas no inequalities were found among boys in 2022. Similar patterns were observed for other illicit drugs; absolute inequalities slightly increased among girls, while relative inequalities decreased among boys.

### Alcohol intoxication

Our findings of stable trends in alcohol intoxication across gender and age groups from T1 to T2 align with previous results reported by the ESPAD group [4]. We expected an overall decrease in the prevalence of alcohol intoxication during the pandemic, as adolescent drunkenness is associated with attendance at social gatherings [56]. However, we did not anticipate the widening gender gap. While this prevalence remained stable for upper secondary girls, it slightly increased among the 10th-grade girls. In contrast, we observed a slight decline in alcohol intoxication in both 10th-grade and upper secondary boys during and following the pandemic. Evidence suggests that girls experienced increased levels of psychological distress during and after the pandemic [31–34, 57]. Given that females are more likely to turn to alcohol as coping mechanism for life stressors [58, 59], this may explain the gender differences we observed during these periods. Although the increase in alcohol intoxication among the youngest girls is small, it is still a cause for concern and warrants close monitoring.

Regarding the association between SEP and adolescent drinking patterns, the evidence is mixed, with many studies reporting only a weak or no association [60]. Consistent with Pape and colleagues [23] we found that among 10th-grade adolescents, alcohol intoxication was more prevalent in those with lower SEP compared to their higher SEP peers. Conversely, among upper secondary adolescents, intoxication was more prevalent in those with higher SEP compared to their more disadvantaged peers. A systematic review identified factors such as parental modelling, monitoring, combined with restricting access to alcohol, and fostering a high-quality parent-child relationship as associated with delaying the onset of alcohol use [61]. Pape et al. [23] found that parents with lower levels of education exercised a less stringent parenting style and engaged in less monitoring. They were also more likely to allow their adolescents to drink and to serve them alcohol [23].

In Norway, it is quite common for adolescents to begin drinking alcohol as they approach their mid-teens. By their first year of upper secondary school, nearly half of both boys and girls report having consumed alcohol, and by the third-year upper secondary students, this figure rises to close to 80% [42]. Studies involving adults link high levels of alcohol consumption to higher socioeconomic groups [19]. It is thus possible that adolescents start adopting their parents’ drinking habits at the same time alcohol access becomes easier. Furthermore, a British study found a strong association between increased access to spending money and binge, frequent and public drinking [62]. This might explain the shift in inequality between socioeconomic groups as adolescents age, as identified in our study.

### Cannabis use

Our results indicate an increasing prevalence of cannabis use among both 10th-grade and upper secondary boys and girls over the study period. However, according to Raitasalo and colleagues [63], the proportion of adolescents using only cannabis remains low and stable, but cannabis use is increasingly prevalent among alcohol users. Previous studies have suggested that greater drug availability, a decrease in health risk perception, and changing attitudes towards cannabis use may help explain this rise [4]. Additionally, a recent Norwegian study found that 20% of upper secondary students experienced peer pressure to use cannabis [64]. Shifts towards more liberal cannabis policies in Western countries, combined with debates in Norway related to a drug reforms [65], and a 2022 Supreme Court ruling [66] that exempts individuals with drug addiction from fines for personal drug use, may also be influencing changes in adolescent attitudes toward cannabis use.

In accordance with the ESPAD group [4], we found that boys exhibited a higher likeliehood of past-year cannabis use then girls. The gender differences were most prominent during T2, but the gap has narrowed in subsequent years. Evidence suggests that an equalization of alcohol and drug use has occurred across genders among current adolescent cohorts compared with previous generations [67, 68]. These findings might indicate that shifts in social norms and a changing cultural environment are influencing gender attitudes towards substance use.

Throughout the study period, lower SEP girls reported a higher prevalence of cannabis use compared with their higher SEP peers, a pattern also observed among the youngest boys. Additionally, socioeconomic inequalities in cannabis use widened between 2014 and 2022 among girls attending upper secondary schools. Early onset of cannabis use is associated with heavier use in later life [69]. However, according to von Sydow et al. [70], socioeconomic background is not a strong predictor of lifetime cannabis use, though a less privileged background does increase the risk of substance abuse and dependence. An examination of users’ influences from, and interactions with, their social environments is key to our understanding of substance use patterns across a population’s socioeconomic groups [71, 72].

Similar to Sandøy [73], we observed a small decline in cannabis use during the pandemic, primarily among boys. Although socioeconomic inequalities among upper secondary school boys are generally small, a shift in usage patterns was evident over the study period. Before the pandemic, boys from more affluent backgrounds reported higher levels of cannabis use compared to their lower SEP peers. However, by T4, no inequalities were observed between higher and lower SEP boys. While a decrease in cannabis availability during the pandemic has been reported, this was limited to the early lockdown period [74]. One possible explanation is that adolescents spent more time at home with their parents rather than with peers, reducing opportunities for cannabis use. Despite the decline observed during T3, the post-pandemic survey shows a notable increase in cannabis use, with levels in 2022 reaching the highest recorded throughout the study period among girls.

### Other illicit drug use

Similar to cannabis, we observed a small increase in prevalence of other illicit drugs use across genders and age groups throughout the study period. Cocaine, ecstasy/MDMA, amphetamine and prescription medication have become more accessible among adolescents in recent years [4, 12]. This increase appears to be part of a prolonged trend rather than directly related to the COVID-19 pandemic. However, evidence suggests that pandemic measures may have influenced both the availability of illegal drugs and substance use behaviors among young people [75]. Additionally, social distancing measures may have impacted drug dealing practices, leading to a greater use of encrypted messaging services, social media, online sources, and mail and home delivery services [12, 74]. These developments raise concerns about the potential long-term effects of the pandemic on drug usage patterns and the rise of a digital drug market providing easy access to new and more potent substances.

Similarly to cannabis, a larger proportion of boys reported using other illicit drugs compared to their female peers. The explanations for these gender differences are complex. While the risk factors are similar, evidence suggest that gender affects how peer and social relations influence substance use [76]. Boys and girls may differ in their exposure to and response to factors such as relationship with close family and peers.

A higher prevalence of illicit drug use was observed among less affluent adolescents of both genders. However, socioeconomic inequalities among older adolescents slightly decreased during the study period. Substance use is sensitive to price, with consumption generally decreasing as prices rise [24, 77]. Globally, the cocaine market is booming [78], and cocaine is the second most commonly used illicit drug in Europe [12]. A recent Norwegian study found stable patterns in MDMA/ecstasy and amphetamines use since 2018, while other synthetic drugs use has increased [64]. The study also identified cocaine as the most used illicit drug among boys attending their third year of upper secondary school. Given that cocaine is an expensive and often associated with socioeconomically privileged groups, this may explain the increase in SEP inequalities.

### Strengths and limitations

The strengths of this study include the use of a large sample, enabling us to examine overall trends and socioeconomic inequalities in adolescent substance use on a nationwide scale. Adolescent SEPs were assessed using the validated FAS II measure [49] as part of a standardized approach. We calculated both absolute and relative inequality measures, acknowledging that each method carries its own implicit value judgements [79].

Several limitations must be acknowledged. Firstly, it was not possible to combine data over a three-year period for T3 and T4 (as we did for T1 and T2) to include most municipalities at each time period. This was due to the special circumstances during which these data were obtained (i.e., during and after the COVID-19 pandemic), thus there is a risk for selection bias at T3 and T4. For example, there are fewer adolescents residing in major municipalities at T4 compared to other time periods. Given that living in major cities is associated with higher risk of substance use [52], it is likely that the use of cannabis and other illicit drugs is underestimated at T4. To address this, we performed stratified analyses for adolescents residing in major municipalities and those in more sparsely populated areas. Results show, as expected, higher prevalences in use of all substances among adolescents living in major cities. However, patterns of time trends (i.e., stable alcohol intoxication prevalence and rising prevalence of cannabis and other illicit drug use) are similar for adolescents regardless of their municipality of residence.

Secondly, although overall response rates were high, about 30% of the invited students did not participate, introduces potential non-participation bias. Additionally, 10% of the sample had missing data, which were excluded from the analyses. In protect respondents’ anonymity, municipalities with small populations often omitted sociodemographic questions. Thirdly, the sample was underrepresented in terms of upper secondary third-year students and some second-year students, as some upper secondary schools only offered their youngest students the opportunity to participate in the surveys [48]. Additionally, many third-year upper secondary students are vocational apprentices and thus not invited to participate in the survey. Furthermore, voluntary drop-out rates increase with age in upper secondary education, leading to a more homogenous group of students compared with lower grade levels. Since illicit drug use increases with age [42], it is possible that illicit drug use among upper secondary adolescents is underestimated in the present study. Finally, reliance on self-reporting data may result in underreporting of cannabis and other illicit drug due to illegal and stigmatizing nature of these behaviors.

## Implications and conclusions

Our results indicate an increase in cannabis and other illicit drugs use among Norwegian adolescents, across both genders and age groups from 2014 to 2022. Boys were more likely than girls to use cannabis or other illicit drugs, but the gender gap in cannabis use narrowed during the study period. Among 10th-grade adolescents, past-year substance use was higher among those from less affluent backgrounds, although the results were more mixed for students attending upper secondary schools. Stable trends in alcohol intoxication across genders and age groups were observed from 2014 to 2019, aligning with previous ESPAD findings. Contrary to expectations, alcohol intoxication did not decrease during the pandemic; instead, it increased among 10th-grade girls while remaining stable among upper secondary girls. Boys experienced a decline in alcohol intoxication during the pandemic.

These findings emphasize the need of universal intervention measures that encompass the entire adolescent population [80]. It also underpins the importance of monitoring substance use trends at both regional and national levels. Adolescent substance use is linked to experimentation, with few developing dependency or related problems. However, persistent drug use in early years can exacerbate negative psychological, physical, and social outcomes. To effectively address these issues, it is crucial to understand the complex factors influencing substance use patterns, including social, economic, and cultural changes.

Future research should focus on differentiating the impacts of various illicit drugs, including both emerging substances and traditional drugs. Detailed studies on these substances will help in identifying specific risk factors and consequences, leading to more targeted prevention and intervention strategies. Incorporating a comprehensive approach that considers structural, social, and economic changes, along with trends influenced by recent global events, will enhance the development of effective and timely interventions.

## Electronic supplementary material

Below is the link to the electronic supplementary material.

Supplementary Material 1

## Data Availability

The data and materials obtained from the Ungdata surveys are available in a national database administered by Norwegian Social Research (NOVA) and can be found at https://ungdata.no. All data are available on application for research purposes.
