# Supplementary material for: Trends in Norwegian adolescents’ substance use between 2014 and 2022: socioeconomic and gender differences
Source: BMC Public Health. 2024 Sep 12;24:2482. doi: 10.1186/s12889-024-19983-9 (PMC11391704; doi:10.1186/s12889-024-19983-9)
Supplement: Supplementary file 1 — Supplementary Material 1 [file 12889_2024_19983_MOESM1_ESM.pdf]

Supplementary materials

Table S1. Gender differences in past year alcohol intoxication, cannabis use and use of other illicit drugs* among 10th-grade adolescents

	**Alcohol intoxication**			**Cannabis use**			**Use of other illicit drugs**		
	OR	95% CI		OR	95% CI		OR	95% CI	
Girls									
T1 (2014-16)	1.18	1.12	1.23	0.57	0.52	0.64			
T2 (2017-19)	1.08	1.03	1.12	0.54	0.50	0.58			
T3 (2021)	1.30	1.22	1.38	0.60	0.53	0.67	0.60	0.53	0.69
T4 (2022)	1.50	1.41	1.61	0.74	0.66	0.83	0.74	0.66	0.83
time	0.95	0.93	0.97	1.14	1.10	1.18	1.14	1.10	1.18
Interaction girls#time									
female#c.time	1.09	1.06	1.13	1.08	1.02	1.15	1.08	1.02	1.15
p-value	p<0.001			p=0.012			p=0.012		

*Adjusted for differences in the respondents' school year

Table S2. Gender differences in past year alcohol intoxication, cannabis use and use of other illicit drugs* among upper secondary school adolescents

	**Alcohol intoxication**			**Cannabis use**			**Use of other illicit drugs**		
	OR	95% CI		OR	95% CI		OR	95% CI	
Girls									
T1 (2014-16)	1.15	1.11	1.19	0.61	0.58	0.64	0.55	0.48	0.63
T2 (2017-19)	1.03	1.01	1.05	0.51	0.50	0.53	0.43	0.39	0.46
T3 (2021)	1.23	1.18	1.27	0.63	0.59	0.66	0.43	0.39	0.48
T4 (2022)	1.29	1.23	1.34	0.67	0.64	0.71	0.50	0.45	0.56
time	0.96	0.95	0.98	1.11	1.09	1.13	1.49	1.43	1.55
Interaction girls#time									
female#c.time	1.06	1.04	1.08	1.04	1.01	1.08	0.96	0.90	1.03
p-value	p<0.001			p=0.007			p=0.270		

*Adjusted for differences in the respondents' school year

Table S3: Prevalence (%), absolute and relative inequalities in past-year alcohol intoxication, cannabis use and use of other illicit drugs* among boys in 10th-grade residing in less populated municipalities between 2014 and 2022 in the Ungdata survey.

	**Alcohol intoxication** (n=13,274)			**Cannabis use** (n=13,270)			**Other illicit drugs** (n=6,515)		
	coeff.	95% CI		coeff.	95% CI		coeff.	95% CI	
		lower	upper		lower	upper		lower	upper
**Prevalence**									
T1 (2014-2016)	22.3	21.6	23.0	5.0	4.6	5.3			
T2 (2017-2019)	23.4	22.8	24.0	6.8	6.4	7.1			
T3 (2021)	22.2	21.3	23.1	5.4	6.4	5.9	2.0	1.7	2.3
T4 (2022)	19.9	19.1	20.8	7.3	6.7	7.8	2.4	2.1	2.7
p-value for trend	p<0.001			p<0.001			p=0.104		
Relative index of inequality[a]									
T1 (2014-2016)	1.43	1.28	1.59	1.78	1.38	2.30			
T2 (2017-2019)	1.27	1.16	1.39	1.57	1.30	1.90			
T3 (2021)	1.51	1.31	1.75	2.47	1.77	3.45	3.41	1.93	6.02
T4 (2022)	1.44	1.23	1.69	1.95	1.46	2.60	2.69	1.59	4.54
p-value for trend	p=0.558			p=0.230			p= 0.545		
Slope index of inequality[b]									
T1 (2014-2016)	0.08	0.05	0.10	0.03	0.01	0.04			
T2 (2017-2019)	0.05	0.03	0.08	0.03	0.02	0.04			
T3 (2021)	0.09	0.06	0.12	0.04	0.03	0.06	0.02	0.01	0.03
T4 (2022)	0.07	0.04	0.10	0.05	0.03	0.07	0.02	0.01	0.03
p-value for trend	p= 0.820			p=0.021			p=0.890		

Data are regression-based predicted means (95% CI)* adjusted for differences in the respondents' school year ([a]) represents the prevalence-ratio for substance use between the lowest and highest ranked families. ([b]) represents the risk difference for substance use between the least and most affluent families.

Table S4: Prevalence (%), absolute and relative inequalities in past-year alcohol intoxication, cannabis use and use of other illicit drugs* among boys in 10th-grade residing in major municipalities between 2014 and 2022 in the Ungdata survey.

	**Alcohol intoxication** (n=30,371)			**Cannabis use** (n=138,216)			**Other illicit drugs** (n=132,429)		
	coeff.	95% CI		coeff.	95% CI		coeff.	95% CI	
		lower	upper		lower	upper		lower	upper
**Prevalence**									
T1 (2014-2016)	25.4	23.8	27.0	8.6	7.6	9.6			
T2 (2017-2019)	24.5	23.4	25.7	12.4	11.5	13.3			
T3 (2021)	20.0	18.6	21.3	10.1	9.1	11.1	2.9	2.4	3.5
T4 (2022)	25.5	23.2	27.8	13.10	11.3	14.9	3.2	2.3	4.2
p-value for trend	p=0.005			p=0.005			p=0.022		
Relative index of inequality[a]									
T1 (2014-2016)	0.53	0.42	0.67	1.63	1.07	2.49			
T2 (2017-2019)	0.72	0.61	0.85	1.45	1.14	1.85			
T3 (2021)	0.62	0.49	0.78	1.21	0.87	1.69	2.45	1.28	4.67
T4 (2022)	0.82	0.58	1.15	2.58	1.60	4.19	1.58	0.56	4.51
p-value for trend	p=0.125			p= 0.436			p=0.488		
Slope index of inequality[b]									
T1 (2014-2016)	-0.15	-0.21	-0.10	0.04	0.00	0.07			
T2 (2017-2019)	-0.08	-0.12	-0.04	0.04	0.01	0.07			
T3 (2021)	-0.10	-0.14	-0.05	0.02	-0.01	0.05	0.02	0.00	0.04
T4 (2022)	-0.05	-0.13	0.03	0.13	0.07	0.20	0.02	-0.02	0.05
p-value for trend	p=0.078			p= 0.378			p= 0.724		

Data are regression-based predicted means (95% CI)* adjusted for differences in the respondents' school year ([a]) represents the prevalence-ratio for substance use between the lowest and highest ranked families. ([b]) represents the risk difference for substance use between the least and most affluent families.

Table S5: Prevalence (%), absolute and relative inequalities in past-year alcohol intoxication, cannabis use and use of other illicit drugs* among 10th-grade girls residing in less populated municipalities between 2014 and 2022 in the Ungdata survey.

	**Alcohol intoxication** (n= 51,176)			**Cannabis use** (n=51,121)			**Other illicit drugs** (n=16,779)		
	coeff.	95% CI lower	upper	coeff.	95% CI lower	upper	coeff.	95% CI lower	upper
**Prevalence**									
T1 (2014-2016)	25.4	24.7	26.1	3.0	2.7	3.3			
T2 (2017-2019)	24.4	23.8	25.0	3.9	3.6	4.1			
T3 (2021)	26.5	25.6	27.4	3.2	2.8	3.6	1.2	1.0	1.4
T4 (2022)	27.2	26.3	28.2	5.7	5.2	6.2	1.7	1.4	2.0
p-value for trend	p<0.001			p<0.001			p= 0.004		
Relative index of inequality[a]									
T1 (2014-2016)	1.65	1.49	1.82	4.35	3.07	6.15			
T2 (2017-2019)	1.51	1.38	1.65	3.47	2.68	4.50			
T3 (2021)	1.30	1.15	1.47	2.80	1.84	4.26	1.96	0.98	3.90
T4 (2022)	1.37	1.20	1.55	2.60	1.88	3.60	3.80	2.06	7.02
p-value for trend	p= 0.004			p= 0.025			p=0.158		
Slope index of inequality[b]									
T1 (2014-2016)	0.12	0.10	0.15	0.04	0.03	0.05			
T2 (2017-2019)	0.10	0.08	0.12	0.04	0.03	0.05			
T3 (2021)	0.07	0.04	0.10	0.03	0.02	0.04	0.01	0.00	0.01
T4 (2022)	0.09	0.05	0.12	0.05	0.03	0.07	0.02	0.01	0.03
p-value for trend	p= 0.018			p=0.787			p=0.044		

Data are regression-based predicted means (95% CI)* adjusted for differences in the respondents' school year ([a]) represents the prevalence-ratio for substance use between the lowest and highest ranked families. ([b]) represents the risk difference for substance use between the least and most affluent families.

Table S6: Prevalence (%), absolute and relative inequalities in past-year alcohol intoxication, cannabis use and use of other illicit drugs* among 10th-grade girls residing in major municipalities between 2014 and 2022 in the Ungdata survey.

	**Alcohol intoxication** (n=13,648)			**Cannabis use** (n=13,631)			**Other illicit drugs** (n=6,802)		
	coeff.	95% CI lower	95% CI upper	coeff.	95% CI lower	95% CI upper	coeff.	95% CI lower	95% CI upper
**Prevalence**									
T1 (2014-2016)	27.6	26.0	29.3	4.6	3.8	5.3			
T2 (2017-2019)	26.7	25.6	27.9	6.4	5.7	7.0			
T3 (2021)	25.3	23.9	26.7	6.2	5.4	7.0	1.9	1.5	2.4
T4 (2022)	33.2	30.7	35.7	7.9	6.5	9.3	1.6	1.0	2.3
p-value for trend	p=0.077			p<0.001			p=0.099		
Relative index of inequality[a]									
T1 (2014-2016)	0.55	0.44	0.68	2.10	1.19	3.68			
T2 (2017-2019)	0.71	0.61	0.82	2.85	2.04	3.99			
T3 (2021)	0.67	0.56	0.81	1.69	1.12	2.55	2.76	1.28	5.94
T4 (2022)	0.79	0.60	1.04	3.91	2.13	7.17	2.81	0.70	11.31
p-value for trend	p=0.069			p=0.436			p= 0.982		
Slope index of inequality[b]									
T1 (2014-2016)	-0.16	-0.22	-0.11	0.03	0.01	0.06			
T2 (2017-2019)	-0.09	-0.13	-0.05	0.06	0.04	0.09			
T3 (2021)	-0.10	-0.15	-0.05	0.03	0.01	0.06	0.02	0.01	0.04
T4 (2022)	-0.08	-0.17	0.01	0.12	0.07	0.18	0.02	-0.01	0.05
p-value for trend	p=0.088			p= 0.112			p=0.849		

Data are regression-based predicted means (95% CI)* adjusted for differences in the respondents' school year ([a]) represents the prevalence-ratio for substance use between the lowest and highest ranked families. ([b]) represents the risk difference for substance use between the least and most affluent families.

Table S7: Prevalence (%), absolute and relative inequalities in past-year alcohol intoxication, cannabis use and use of other illicit drugs* among boys attending upper secondary education residing in less populated municipalities between 2014 and 2022 in the Ungdata survey.

	**Alcohol intoxication** (n=103,668)			**Cannabis use** (n=103,593)			**Other illicit drugs** (n=103,000)		
	coeff.	95% CI		coeff.	95% CI		coeff.	95% CI	
		lower	upper		lower	upper		lower	upper
**Prevalence**									
T1 (2014-2016)	55.4	54.8	56.0	11.0	10.6	11.4	1.9	1.7	2.1
T2 (2017-2019)	56.8	56.3	57.2	15.7	15.3	16.0	3.5	3.3	3.7
T3 (2021)	55.5	54.8	56.1	13.4	12.9	13.9	4.1	3.8	4.3
T4 (2022)	52.4	51.6	53.1	16.2	15.7	16.8	4.8	4.4	5.1
p-value for trend	p<0.001			p<0.001			p<0.001		
Relative index of inequality[a*]									
T1 (2014-2016)	1.03	0.99	1.07	1.20	1.05	1.37	2.57	1.82	3.63
T2 (2017-2019)	0.97	0.95	1.00	1.03	0.96	1.12	1.54	1.29	1.84
T3 (2021)	1.05	1.00	1.09	1.17	1.02	1.33	1.84	1.42	2.38
T4 (2022)	0.97	0.93	1.02	1.22	1.07	1.38	1.34	1.04	1.72
p-value for trend	p= 0.558			p=0.308			p=0.028		
Slope index of inequality[b]									
T1 (2014-2016)	0.03	0.01	0.05	0.02	0.01	0.04	0.02	0.01	0.02
T2 (2017-2019)	-0.01	-0.02	0.01	0.01	-0.01	0.02	0.02	0.01	0.02
T3 (2021)	0.04	0.01	0.06	0.02	0.00	0.04	0.02	0.01	0.03
T4 (2022)	-0.01	-0.03	0.02	0.03	0.01	0.05	0.02	0.01	0.03
p-value for trend	p=0.437			p= 0.469			p=0.519		

Data are regression-based predicted means (95% CI)* adjusted for differences in the respondents' school year ([a]) represents the prevalence-ratio for substance use between the lowest and highest ranked families. ([b]) represents the risk difference for substance use between the least and most affluent families.

Table S8: Prevalence (%), absolute and relative inequalities in past-year alcohol intoxication, cannabis use and use of other illicit drugs* boys attending upper secondary education residing in major municipalities between 2014 and 2022 in the Ungdata survey.

	**Alcohol intoxication** (n=26,703)			**Cannabis use** (n=26,710)			**Other illicit drugs** (n=22,038)		
	coeff.	95% CI		coeff.	95% CI		coeff.	95% CI	
		lower	upper		lower	upper		lower	upper
Prevalence									
T1 (2014-2016)	54.8	53.6	56.1	22.2	21.1	23.2	2.2	1.8	2.6
T2 (2017-2019)	58.7	57.9	59.6	26.9	26.2	27.7	4.8	4.3	5.2
T3 (2021)	49.6	48.4	50.9	24.3	23.2	25.5	5.1	4.5	5.6
T4 (2022)	58.7	56.9	60.5	26.2	24.6	27.9	7.8	6.8	8.8
p-value for trend	p=0.060			p=0.004			p<0.001		
Relative index of inequality[a1]									
T1 (2014-2016)	0.49	0.45	0.53	0.66	0.56	0.78	1.25	0.71	2.20
T2 (2017-2019)	0.65	0.61	0.68	0.75	0.68	0.83	1.45	1.03	2.05
T3 (2021)	0.58	0.52	0.64	0.79	0.67	0.93	0.89	0.59	1.33
T4 (2022)	0.77	0.68	0.88	1.12	0.88	1.41	1.61	1.00	2.59
p-value for trend	p= 0.125			p= 0.001			p=0.986		
Slope index of inequality[b]									
T1 (2014-2016)	-0.39	-0.42	-0.35	-0.08	-0.12	-0.05	0.00	-0.01	0.02
T2 (2017-2019)	-0.24	-0.27	-0.22	-0.07	-0.10	-0.05	0.02	0.01	0.04
T3 (2021)	-0.27	-0.31	-0.22	-0.06	-0.10	-0.02	0.00	-0.02	0.02
T4 (2022)	-0.14	-0.20	-0.07	0.03	-0.04	0.09	0.03	0.00	0.07
p-value for trend	p<0.001			p=0.007			0.426		

Data are regression-based predicted means (95% CI)* adjusted for differences in the respondents' school year ([a]) represents the prevalence-ratio for substance use between the lowest and highest ranked families. ([b]) represents the risk difference for substance use between the least and most affluent families.[1]convergence not achieved in adjusted analyses, results of unadjusted analyses are presented

Table S9: Prevalence (%), absolute and relative inequalities in past-year alcohol intoxication, cannabis use and use of other illicit drugs* among girls attending upper secondary education residing in less populated municipalities between 2014 and 2022 in the Ungdata survey.

	**Alcohol intoxication** (n=107,829)			**Cannabis use** (n=107,835)			**Other illicit drugs** (n=107,396)		
	coeff.	95% CI		coeff.	95% CI		coeff.	95% CI	
		lower	upper		lower	upper		lower	upper
**Prevalence**									
T1 (2014-2016)	60.1	59.5	60.7	7.0	6.7	7.3	1.0	0.9	1.2
T2 (2017-2019)	59.1	58.6	59.5	8.9	8.7	9.2	1.6	1.5	1.7
T3 (2021)	61.3	60.6	61.9	9.0	8.6	9.4	1.8	1.6	2.0
T4 (2022)	59.7	59.0	60.5	12.2	11.7	12.7	2.6	2.4	2.9
p-value for trend	p= 0.201			p<0.001			p<0.001		
Relative index of inequality[a]									
T1 (2014-2016)	1.07	1.03	1.11	1.37	1.16	1.62	2.82	1.79	4.47
T2 (2017-2019)	1.03	1.00	1.05	1.39	1.25	1.54	2.27	1.74	2.97
T3 (2021)	1.03	0.99	1.06	1.80	1.54	2.11	3.59	2.45	5.25
T4 (2022)	0.96	0.92	0.99	1.58	1.37	1.82	2.22	1.59	3.09
p-value for trend	p= 0.004			p=0.043			p=0.833		
Slope index of inequality[b]									
T1 (2014-2016)	0.05	0.03	0.07	0.03	0.02	0.04	0.01	0.01	0.01
T2 (2017-2019)	0.03	0.01	0.04	0.03	0.02	0.04	0.01	0.01	0.02
T3 (2021)	0.03	0.01	0.05	0.05	0.04	0.07	0.02	0.02	0.03
T4 (2022)	-0.02	-0.05	0.00	0.06	0.04	0.08	0.02	0.01	0.03
p-value for trend	p<0.001			p<0.001			p<0.001		

Data are regression-based predicted means (95% CI)* adjusted for differences in the respondents' school year ([a]) represents the prevalence-ratio for substance use between the lowest and highest ranked families. ([b]) represents the risk difference for substance use between the least and most affluent families.

Table S10: Prevalence (%), absolute and relative inequalities in past-year alcohol intoxication, cannabis use and use of other illicit drugs* among girls attending upper secondary education residing in major municipalities between 2014 and 2022 in the Ungdata survey.

	Alcohol intoxication (n=30,371)			Cannabis use (n=30,381)			Other illicit drugs (n=25,033)		
	coeff.	95% CI		coeff.	95% CI		coeff.	95% CI	
		lower	upper		lower	upper		lower	upper
**Prevalence**									
T1 (2014-2016)	57.7	56.6	58.9	15.4	14.5	16.2	1.3	1.0	1.6
T2 (2017-2019)	59.4	58.6	60.2	15.8	15.2	16.4	1.9	1.6	2.1
T3 (2021)	56.6	55.4	57.8	16.9	16.0	17.8	2.5	2.1	2.9
T4 (2022)	63.7	62.0	65.4	16.3	14.9	17.6	3.3	2.7	4.0
p-value for trend	p=0.003			p= 0.036			p<0.001		
Relative index of inequality[a1]									
T1 (2014-2016)	0.48	0.45	0.52	0.75	0.62	0.90	3.11	1.57	6.16
T2 (2017-2019)	0.62	0.59	0.65	0.93	0.81	1.05	2.58	1.54	4.33
T3 (2021)	0.62	0.57	0.66	0.93	0.77	1.11	1.26	0.76	2.11
T4 (2022)	0.75	0.67	0.83	1.68	1.27	2.22	3.13	1.62	6.05
p-value for trend	p<0.001			p=0.001			p=0.456		
Slope index of inequality[b]									
T1 (2014-2016)	-0.45	-0.48	-0.41	-0.04	-0.07	-0.01	0.02	0.01	0.02
T2 (2017-2019)	-0.29	-0.31	-0.26	0.00	-0.02	0.02	0.02	0.01	0.03
T3 (2021)	-0.29	-0.33	-0.25	-0.01	-0.04	0.02	0.01	-0.01	0.02
T4 (2022)	-0.18	-0.24	-0.12	0.08	0.04	0.13	0.04	0.02	0.06
p-value for trend	p<0.001			p<0.001			p=0.616		

Data are regression-based predicted means (95% CI)* adjusted for differences in the respondents' school year ([a]) represents the prevalence-ratio for substance use between the lowest and highest ranked families. ([b]) represents the risk difference for substance use between the least and most affluent families.[1]convergence not achieved in adjusted analyses, results of unadjusted analyses are presented
